# Comparing the toxicity of selected plant extract anthelmintics to levamisole hydrochloride and piperazine citrate in chickens

**DOI:** 10.4102/ojvr.v92i1.2212

**Published:** 2025-09-10

**Authors:** Gerald Zirintunda, John Kateregga, Agnes Sarah Nalule, Patrick Vudriko, Savino Biryomumaisho, James Okwee-Acai

**Affiliations:** 1 Department of Veterinary Pharmacy and Comparative Medicine, College of Veterinary Medicine and Biosecurity, Makerere University, Kampala, Uganda; 2 Department of Animal Production and Management, Faculty of Agriculture and Animal Sciences, Busitema University, Tororo, Uganda

**Keywords:** extract, herbal, synthetic, toxicity, eosinophilia, sodium, lesions, kidney, liver

## Abstract

In a controlled experiment, we compared renal-hepatal, haematological and organ histopathology responses in chickens treated with Crude extracts of *Capsicum annuum* L. and *Carica papaya* L. to those treated with synthetic anthelmintics. Twenty-one indigenous seven-week-old chickens were fed on Nuvita^®^ feeds Uganda limited and adlib tap municipal water. They were divided into seven groups of three chickens per group. The treatments were; CPLe (*Carica papaya* L. ethanol extract), CPLa (*Carica papaya* L. acetone extract), CAFe (*Capsicum annuum* L. ethanol extract), CAFa (*Capsicum annuum* L. acetone extract), levamisole, piperazine and phosphate buffered saline (PBS). CPLe, CPLa, CAFe, CAFa were given at a dose of 0.48 g per bird (1.37 g/kg body weight) as determined from a previous in-vitro experiment. Levamisole hydrochloride was given at 25 mg/kg body weight and piperazine citrate at 100 mg/kg body weight. The control group received 0.2% dimethyl sulfoxide (DMSO) in PBS. The treatments were individually administered orally and repeated on the second day. The birds were bled and sacrificed one week after treatment. Blood was submitted for haematology and harvesting of serum for renal-hepatal function tests. The heart, kidneys and liver were also harvested for histological examinations. CPLe caused significantly raised albumin compared to CAFe (*p* = 0.02), levamisole caused significantly raised AST compared to CAFe (*p* = 0.04). All extracts and synthetic anthelmentics increased the number of eosinophils, indicating an increased inflammatory response. CPLa, CAFe, CAFa and levamisole were toxic to the kidneys. All extracts were toxic to the liver except CPLe. Piperazine and levamisole were as well toxic to the liver.

**Contribution:**

Plant extracts are not necessarily safer than synthetic anthelmintics and should be used with caution.

## Introduction

Synthetic and natural therapeutic products can both induce toxicities, especially when administered at very high doses (Abdel-Daim et al. 2018). Over the years, natural products, including those from plants, have been used to manage several conditions in animals. Most (81%) of the plant extracts were reported to have toxic effects in various animal tissues, unless dosed prudently (Viegi & Vangelisti 2011). Although many plants have been evaluated for their ethnoveterinary potential, few of them have been assessed for toxicity (Habeeb 2010; McGaw & Eloff 2008; Sunder 2016).

Previous studies determined the cytotoxic effects of several plant extracts on parasitic agents but have been silent on their effects on the hosts. Grades of toxicity may vary for the same plant from different geographical locations because of the possible differences in levels of soil minerals and contaminants (González-Miqueo et al., 2010). Plants accumulate toxic materials, especially in areas of high environmental toxicants (Ssempijja et al. 2020). Many ethnomedicinal plants are known to contain unacceptable levels of heavy metals (Kasozi et al. 2021). High levels of lead and zinc in plants predispose to renal diseases (Wismer 2015). Some plants even containing negligible levels of heavy metals have been associated with nephrotoxicity for unknown reasons (Baudoux et al. 2017).

Hypovitaminosis A in animals has been associated with renal diseases (Cojean, Larrat & Vergneau-Grosset 2020), while hypervitaminosis D_3_ also causes renal diseases. High vitamin C concentration increases iron uptake, which predisposes animals to renal diseases. Damage to the kidney of any cause manifests with changes in the concentrations of blood urea nitrogen (BUN), creatinine, uric acid and changes in serum osmolality (Gounden, Bhatt & Jialal 2021; Kluwe 1981). Changes in kidney weight and histopathological changes are also plausible indicators of nephrotoxicity in laboratory animals. Although piperazine citrate was said to have wide safety margin, its toxicity manifests as swollen kidney, enlarged ureters and visceral gout (Shumard 1956). Levamisole is said to increase plasma creatinine levels in other species, but it is not known whether this happens even in chicken. The renal function effects of anthelmintics in chicken are not well known.

Haematological parameters are affected by animal physiological condition, genotype, age, sex, nutrition, climatic conditions and prevailing pathology. The effects of piperazine citrate on haematological parameters of chicken are not well known. The effects of levamisole hydrochloride on haematological parameters depend on the number of given doses: a single dose increases the parameters, while triple doses decrease the parameters (Kuropka et al. 2022).

There is no information on haematology of indigenous chicken of Uganda when exposed to therapeutic dozes of plant extracts compared to that of commercial dewormers for nematodes (levamisole and piperazine citrate). The effect of ethnoveterinary practices (EVPs) on the haematological parameters of chicken has not been widely documented. This is the first study in Uganda reporting the effects of *Carica papaya* L. and *Capsicum annuum* L. on chicken haematological parameters.

Various plant extracts and commercial drugs have been shown to have toxic effects on the cardiovascular system of animals. Some of the pathologic effects of chemotherapeutic agents and plant extracts include, but are not limited to foci of haemorrhages on various organs, myocardial changes, vascular damage and necrosis of myocardium. Other effects may include: acute inflammatory response, fibrinous or oedematous exudates in various tissues including cardiac muscle fibres. Toxic substances may cause congestion of coronary vessels (Lateif et al. 2024).

Hepatoxicity may manifest as hepatomegaly, presence of haemorrhages, focal necrosis, discolorations, accumulation of lipids in hepatocytes, jaundice, biliary tissue proliferation, focal fibrosis and hyperplasia (Sathiyanarayanan & Arulmozhi 2007).

Piperazine and levamisole are known to remain as drug residues in the tissues of chickens, but the organ-toxic effects in chickens have not been progressively assessed (El-Kholy & Kemppainen 2005; Ke, Chen & Lin 2024). Levamisole acute toxicity has been reported in mice (Almawla & Al Baggou 2023), but extensive toxicity studies have not been appraised in chickens.

The use of EVPs has been studied in other species and cause various complications, but such studies have not been conducted in chickens. The effect of EVP on the immune system, nutritional status and metabolism of chicken is not known. The rising campaign for EVP alternatives in chickens requires a concomitant appraisal of their effects on body systems. This study intended to compare the toxicological indices of ethno-anthelmintics (*C. annuum* L. and *C. papaya* L.) to the commonly used synthetic anthelmintics (piperazine citrate and levamisole hydrochloride). The indices considered here are haematology, renal functions, liver functions and selected organ histopathology.

## Research methods and design

### Experimental design

The design was adopted with modifications from the National Research Council (NRC 2006). An experimental design to study the safety of *C. papaya* L. leaves extract, *C. annuum* L. fruits extract, piperazine citrate and levamisole hydrochloride chicken anthelmintics was conducted by comparing selected organ histo-toxicology, hepato-renal functional tests and haematological parameters. A cohort of indigenous chicks (*Gallus gallus domesticus*) in the Soroti district (Uganda) were left to scavenge with flocks for 6 weeks before being selected for caging in the 7th week. In the 7th week, which was also the acclimatisation week, the chickens were fed on standard broiler finisher from Nuvita feeds (Uganda Millers Limited^®^), and *ad libitum* tap water was provided. In the 8th week, the chickens were subjected to treatments on 6th and 7th May 2024. The chickens from the different groups were then transported to the College of Veterinary Medicine post-mortem laboratory, where whole blood samples were collected. Chickens were then euthanised, and organs (liver, heart and kidneys) were harvested.

### Climatic conditions of Soroti district

Soroti district is located at latitude 1^o^ 42’ 47.4516’’N and longitude 33^o^36’ 22.986’’ E. In May 2024, the temperature ranged from 18.9 °C to 27.1 °C, with an average humidity of 84%. The rainfall was 192 mm (7.56’’), and the ultraviolet (UV) index ranged from 3 mW/m^2^ to 5 mW/m^2^.

### Preparation and storage of plant extracts

Leaves of *C. papaya* L. and fruits of *C. annuum* L. were obtained from Soroti district, Eastern Uganda, during the rainy season (May 2023). The plants were identified and authenticated by senior botanists from the College of Natural Sciences, Makerere University. A conventional Soxhlet extraction method was used to obtain plant extracts (Hirondart et al. 2020). Freshly collected plant material was washed with tap water to remove observable debris. Plant material was air dried in the shade for 2 weeks before grinding to a fine powder using a coffee grinder. Ten grams of ground plant material and 5 g of pumice stones were placed in a cellulose thimble plugged with cotton wool. The setup was placed in the conventional Soxhlet extraction apparatus containing 300 mL of solvent. Extraction was performed using a solid to liquid ratio of 1 to 12 (g/mL) for 8 h. The extraction was done in duplicate using analytical-grade ethanol and acetone as solvents. The *C. papaya* L. ethanol extract was labelled CPLe and the acetone extract CPLa. The *C. annuum* L. ethanol extract was labelled CAFe and the acetone extract CAFa. The extract was concentrated under vacuum and conserved at 4 °C.

### Management of study chickens

Traditionally hatched chicks of indigenous breed were left to scavenge free-range with the hens to expose them to nematodal parasites in the environment. Faecal samples were collected from the chickens and examined for nematode infection in the 7th week. The extent of infection was graded, and chickens with a worm burden of at least 200 eggs per gram of faecal sample and weighing between 300 g and 350 g were selected for the experiments. The chickens were then put into cages in groups of three chickens per cage, with each cage having an area of 0.16 m^2^. They were fed on grower mash from Nuvita feeds (Uganda Millers Limited^®^) and given *ad libitum* tap water. The detail of the composition of the feeds is shown in Table 1. Birds were left to acclimatise in the cages for 1 week before treatments were given. During the free-ranging period, no supplementation was provided; however, the animals received vaccination against Newcastle disease.

**TABLE 1:** Composition of grower finisher feed used in the study.

Ingredient	Composition
**Crude protein (%)**	18
Metabolisable energy (Kcal/kg)	3150
Calcium (%)	0.89
Available phosphorus (%)	0.38
Sodium (%)	0.2
Methionine (%)	0.38
Methionine + cystine (%)	0.75
Lysine (%)	1.0
Threonine (%)	0.55
Tryptophan (%)	0.18
Arginine (%)	1.1
Valine (%)	0.56
Leucine (%)	0.9
Isoleucine (%)	0.55
Histidine (%)	0.28
Phenylalanine (%)	0.6
**Trace minerals (per kg)**	
Manganese (mg)	70
Iron (mg)	20
Copper (mg)	8
Zinc (mg)	70
Iodine (mg)	0.5
Selenium (mg)	0.3
**Vitamins (per kg)**	
Vitamin A (I.U)	8000
Vitamin D_3_ (I.U)	3500
Vitamin E (I.U)	50
Vitamin K (I.U)	3
Thiamine (mg)	4
Riboflavin (mg)	5
Pyridoxine (mg)	4
Pantothenic acid (mg)	14
Folic acid (mg)	1
Biotin (µg)	100
Niacin (mg)	40
Choline (mg)	400
Vitamin B_12_ (µg)	12

Note: Nuvita feeds, Jinja City in Uganda.

### Administration of treatments

Seven separate treatment groups were set up in triplicate: CAFa, CAFe, CPLa, CPLe, piperazine citrate, levamisole hydrochloride and 0.2% DMSO. The lowest concentration of compound that paralysed the highest number of *Ascaridia galli* worms in the *in vitro* experiments (0.08 g/mL) was doubled to determine the concentration of the extracts that were used in chickens (Zirintunda et al. 2025). This was to simulate the discriminating concentration concept. The extracts were administered in 3 mL volumes (0.48 g) per chicken per day. Piperazine citrate was given at 100 mg/kg body weight as recommended by the manufacturer and levamisole hydrochloride at 25 mg/kg body weight also as recommended by the manufacturer. DMSO (0.2%) in phosphate-buffered saline (PBS) was administered as the negative control. The treatment was repeated on the following day. Birds were sacrificed a week after treatments in the post-mortem room of the College of Veterinary Medicine, Makerere University.

### Organ collection and histological slide preparation

The chickens were euthanised by mechanical cervical dislocation (Boyal et al. 2020). Chickens were sacrificed by severing the cervical region using a surgical blade. Post-mortems were conducted, and the target organs (heart, kidneys and liver) were collected in 10% neutral buffered formalin solution for 48 h. The fixed specimens were trimmed and placed in labelled cassettes, then placed in formalin again and finally transferred into the tissue processor. The tissues were processed in a semi-automated processor, based on an established procedure (Dey 2018).

### Measurement of hepato-renal function

Blood urea nitrogen, sodium (Na^+^), chloride (Cl^-^) and creatinine (Cre) were determined using a semi-automatic clinical analyser, DIRUI-7000 (Jilin Jingquan Medical Equipment Limited^®^, Changchun, China). Uric acid and the liver function parameters – aspartate aminotransferase (AST), alanine transaminase (ALT), glutamyl transferase (GT), total bilirubin (TB), direct bilirubin (DB), total proteins (TPs), albumin (ALBU) and globulins (Globu) – were determined using Cobas^®^ C311 analyser (Roche, Germany).

### Measurement of haematological and blood parameters

Blood was collected by jugular puncture (Kelly & Alworth 2013) into pink top tubes containing ethylenediaminetetraacetic acid (EDTA). Gentle inversion was done to mix the blood with the anticoagulant, and processing for haematological analysis was conducted immediately.

Manual blood smear review (MBSR) and manual differential leukocyte counts (MDLCs) were done as described by Comar, Malvezzi and Pasquini (2017). Meticulous examination of well-prepared stained blood smears with keen assessment of any morphological changes was conducted by a team having 10 years to 40 years’ experience in MBSR, in the central diagnostic laboratory of the College of Veterinary Medicine and Biosecurity, Makerere University.

### Statistical analysis

Data were entered in Microsoft (MS) Excel for cleaning and transferred to SPSS^®^ version 26 for analysis. Descriptive statistics were done to obtain the mean, median, standard deviation and confidence intervals. One-way analyses of variance (ANOVA) was performed, followed by Tukey’s honest significant difference (HSD) multiple comparison. Statistical significance was considered at *p* ≤ 0.05.

### Toxicological assessment

The protocols were adopted with modifications from the Organisation of Economic Cooperation Development (OECD 1996). The renal function tests, liver function tests and the haematology parameters of the treatments were compared to their control; a treatment having one or more aspects significantly different was considered toxic. The significance levels and the number of significant aspects showed the toxicity levels. For organ histopathology, the heart, kidney and liver of the three birds per treatment were assessed. The number of organs with observable lesions over the total number of organs per treatment was the scoring method. Having no observable lesions for a treatment (*n* = 0/9) was considered safe, having one organ with observable lesions (*n* = 1/9) was considered moderate and having more than one organ with observable lesions was considered toxic (safe < *n* = 1/9 > toxic).

### Ethical considerations

Ethical clearance to conduct this study was obtained from the Uganda National Council for Science and Technology (UNCST Ref A220ES). The guidelines for research during the coronavirus disease 2019 (COVID-19) pandemic concerning safety of respondents (UNCST 2020) were followed. An ethical review certificate was acquired from the School of Veterinary Medicine and Animal Resources Institutional Review Board (IRB) (SVAR_IACUC/93/2021). Permission to conduct research was also granted by the Soroti District Veterinary Office.

## Results

### Renal function parameters

The results of BUN, sodium, chloride, creatinine and uric acid measurements are shown in Table 2.

**TABLE 2:** Renal function parameters in chickens treated with herbal and synthetic anthelmintics.

Number	Sample identification or treatment group	BUN (mmol/L)	Na (mmol/L)	Cl (mmol/L)	Cre (µmol/L)	Uric acid (mmol/L)
1	CAFa	9.23	16.00	98.5	0.82	0.072
2	CAFa	10.02	15.06	95.2	0.98	0.157
3	CAFa	2.10	15.03	99.5	0.78	0.071
4	CAFe	9.32	25.59	96.6	1.30	0.178
5	CAFe	9.32	30.34	92.9	1.37	0.143
6	CAFe	13.06	27.97	93.8	1.07	0.214
7	CPLa	0.26	27.53	95.1	0.78	0.084
8	CPLa	9.89	25.09	89.5	1.41	0.089
9	CPLa	0.09	20.02	93.4	1.34	0.146
10	CPLe	9.31	19.79	92.0	0.50	0.090
11	CPLe	3.03	27.61	93.7	0.78	0.151
12	CPLe	4.96	26.35	99.8	0.82	0.140
13	Pip	6.53	26.21	95.0	0.57	0.095
14	Pip	6.25	24.16	95.3	1.12	0.077
15	Pip	2.81	23.95	93.3	0.87	0.310
16	Lev	2.10	25.23	97.9	0.99	0.073
17	Lev	5.31	22.03	97.5	0.74	0.181
18	Leva	9.72	25.77	95.1	0.80	0.082
19	PBS	10.05	25.37	96.0	0.73	0.143
20	PBS	3.03	27.73	94.2	0.69	0.182
21	PBS	3.03	17.22	92.7	1.09	0.089

BUN, blood urea; Na, sodium; Cl, chloride; Cr, creatinine; CAFe, *Capsicum annuum* L. ethanol extract; CAFa, *Capsicum annuum* L. acetone extract; CPLe, *Carica papaya* L. ethanol extract; CPLa, *Carica papaya* L. acetone extract; Pip, Piperazine citrate; Lev/Leva, Levamisole hydrochloride; PBS, Phosphate buffered saline.

#### Blood urea nitrogen

There was no significant difference between the different plant extracts and the control treatments. There was no significant difference in the BUN levels for the extracts and piperazine citrate treatments (*p* > 0.05). There was no significant difference in the BUN levels for the extracts and levamisole hydrochloride (*p* > 0.05).

#### Sodium

There was no significant difference between the different plant extracts and the control treatments; however, CAFa extract demonstrated significantly different (lower) levels of sodium compared to CPLe extract (*p* = 0.046). There was no significant difference in the sodium levels for the extracts and piperazine citrate treatments (*p* > 0.05). There was no significant difference in the sodium levels for the extracts and levamisole hydrochloride (*p* > 0.05).

#### Chloride

There was no significant difference between the different plant extracts and the control treatments. There was no significant difference in the chloride levels for the extracts and piperazine citrate treatments (*p* > 0.05). There was no significant difference in the chloride levels for the extracts and levamisole hydrochloride (*p* > 0.05).

#### Creatinine

There was no significant difference between the different plant extracts and the control treatments (Table 3). There was no significant difference in the creatinine levels for the extracts and piperazine citrate treatments (*p* > 0.05). There was no significant difference in the creatinine levels for the extracts and levamisole hydrochloride (*p* > 0.05).

**TABLE 3:** Multiple comparisons of renal functions in chickens treated with herbal and synthetic anthelmintics.

(I) Sample identification	Tukey HSD						
	(J) Sample identification	Mean ± s.d.	Mean difference (I-J)	Std. error	Sig.	95% confidence interval	
						Lower bound	Upper bound
**Dependent variable: BUN**							
Control	CPLa	3.413 ± 5.61	1.95670	3.09937	0.994	−8.62640	12.53970
	CAFa	7.118 ± 4.36	−1.74670	3.09937	0.997	−12.32970	8.83640
	CPLe	5.767 ± 3.23	−0.03967	3.09937	1.000	−10.97970	10.18640
	CAFe	10.567 ± 2.16	−5.19670	3.09937	0.640	−15.77970	5.38640
	Leva	5.710 ± 3.83	−0.34000	3.09937	1.000	−10.92300	10.24300
	Pip	5.197 ± 2.07	0.17330	3.09937	1.000	−10.40970	10.75640
	Control	5.370 ± 4.05					
**Dependent variable: Na**							
CAFa	CPLa	24.213 ± 3.83	−8.85000	2.66019	0.058	−17.93350	0.23350
	Control	23.440 ± 5.51	−8.07670	2.66019	0.097	−17.16010	1.00680
	CPLe	24.583 ± 4.20	−9.22000*	2.66019	0.046*	−18.30350	−0.13650
	CAFe	27.967 ± 2.38	−12.60330*	2.66019	0.005**	−21.68680	−3.51990
	Leva	24.343 ± 2.02	−8.98000	2.66019	0.054	−18.06350	0.10350
	Pip	24.773 ± 1.25	−9.41000*	2.66019	0.040*	−18.49350	−0.32650
	CAFa	15.363 ± 0.55					
Control	CPLa	24.213 ± 3.83	−0.77330	2.66019	1.000	−9.85680	8.31010
	CAFa	15.363 ± 0.55	8.07670	2.66019	0.097	−1.00680	17.16010
	CPLe	24.583 ± 4.20	−1.14330	2.66019	0.999	−10.22680	7.94010
	CAFe	27.967 ± 2.38	−4.52670	2.66019	0.626	−13.61010	4.55680
	Leva	24.343 ± 2.02	−0.90330	2.66019	1.000	−9.98680	8.18010
	Pip	24.773 ± 1.25	−1.33330	2.66019	0.998	−10.41680	7.75010
**Dependent variable: Cl**							
Control	CPLa	92.667 ± 2.87	1.63300	1.95280	0.976	−5.03500	8.30100
	CAFa	97.733 ± 2.25	−3.43300	1.95280	0.593	−10.10100	3.23500
	CPLe	95.167 ± 4.10	−0.86700	1.95280	0.999	−7.53500	5.80100
	CAFe	94.433 ± 1.93	−0.13300	1.95280	1.000	−6.80100	6.53500
	Leva	96.833 ± 1.51	−2.53300	1.95280	0.842	−9.20100	4.13500
	Pip	94.533 ± 1.08	−0.23300	1.95280	1.000	−6.90100	6.43500
	Control	94.300 ± 1.65					
**Dependent variable: Creatinine**							
Control	CPLa	1.177 ± 0.35	−0.34000	0.17644	0.496	−0.94250	0.26250
	CAFa	0.860 ± 0.11	−0.02330	0.17644	1.000	−0.62580	0.57910
	CPLe	0.700 ± 0.17	0.13670	0.17644	0.984	−0.46580	0.73910
	CAFe	1.250 ± 0.16	−0.41000	0.17644	0.298	−1.01250	0.19250
	Leva	0.843 ± 0.13	−0.00670	0.17644	1.000	−0.60910	0.59580
	Pip	0.853 ± 0.28	−0.01670	0.17644	1.000	−0.61910	0.58580
	Control	0.837 ± 0.22					
**Dependent variable: Uric acid**							
Control	CAFa	0.100 ± 0.05	0.03800	0.052116	0.988	−0.13996	0.21596
	CAFe	0.178 ± 0.04	−0.04033	0.052116	0.984	−0.21829	0.13762
	CPLa	0.106 ± 0.04	0.03167	0.052116	0.995	−0.14629	0.20962
	CPLe	0.127 ± 0.03	0.01100	0.052116	1.000	−0.16696	0.18896
	Lev	0.112 ± 0.06	0.02600	0.052116	0.998	−0.15196	0.20396
	Pip	0.161 ± 0.13	−0.02267	0.052116	0.999	−0.20062	0.15529

Note: Control = 0.138 ± 0.05.

BUN, blood urea nitrogen; Na, sodium; CI, chloride; CAFe, *Capsicum annuum* L. ethanol extract; CAFa, *Capsicum annuum* L. acetone extract; CPLe, *Carica papaya* L. ethanol extract; CPLa, *Carica papaya* L. acetone extract; Pip, Piperazine citrate; Lev, Levamisole hydrochloride.

* , *p* < 0.05;

** , *p* < 0.01.

#### Uric acid

There were no significant differences in the *uric acid levels* among the different extracts (*p* > 0.05). There was no significant difference in the uric acid levels for the extracts and piperazine citrate treatments (*p* > 0.05). There was no significant difference in the uric acid levels for the extracts and levamisole hydrochloride (*p* > 0.05).

There was no difference in the effects on renal parameters between the plant extracts (CPLa, CPLe, CAFa and CAFe) and the synthetic anthelmintics (piperazine citrate and levamisole hydrochloride) Table 3 (*p* > 0.05).

There was no significant difference in BUN, sodium, chloride, creatinine and uric acid when comparing the treatments and control birds. There was a significant difference in Na^+^ levels when comparing chickens treated with CAFa and CPLe (*p* = 0.046), CAFe (*p* = 0.005) and piperazine citrate (*p* = 0.04).

### Liver function parameters

The results of the liver function parameters are shown in Table 4. There was no significant difference in the effects on liver function parameters for plant extracts compared to the control (*p* > 0.05). There was no significant difference in the effects on liver function parameters for piperazine citrate compared to the control (*p* > 0.05). There was no significant difference in the effects on liver function parameters for levamisole hydrochloride compared to the control (*p* > 0.05). There was no significant difference between the effects of the various plant extracts on the liver function parameters (*p* > 0.05) except for serum albumin, where CPLe caused significantly higher albumin levels compared to CAFe (*p* = 0.02), see Table 5.

**TABLE 4:** Liver function parameters in chickens treated with herbal and synthetic anthelmintics.

Treatment	AST (IU/L)	ALT (IU/L)	ALP (IU/L)	GT (IU/L)	TB (micro moles/L)	DB (MM/L)	TP (g/L)	ALBU (g/L)	Globu (g/L)
CAFa	248.2	2.7	1505	23	0.3	0.1	43.6	14.0	29.6
CAFa	253.6	3.0	1542	25	0.4	0.2	50.9	12.8	38.1
CAFa	287.7	3.3	944	22	0.2	0.2	54.4	14.1	40.3
CAFe	216.3	3.8	907	14	0.2	0.2	33.9	10.5	23.4
CAFe	305.4	3.1	679	24	0.5	0.1	48.4	13.7	34.7
CAFe	239.3	2.5	937	18	0.4	0.2	50.3	11.2	39.1
CPLa	299.9	2.5	483	18	0.4	0.0	56.8	14.8	42.0
CPLa	309.8	4.0	456	24	0.2	0.0	41.6	12.7	28.9
CPLa	219.3	2.9	1682	17	0.1	0.0	42.7	12.2	29.8
CPLe	299.9	3.3	1431	20	0.2	0.0	52.4	15.1	37.3
CPLe	292.5	2.5	1077	24	0.2	0.2	38.0	15.6	22.4
CPLe	382.2	2.7	1594	27	0.3	0.1	50.6	16.5	34.1
Pip	268.9	1.8	1380	27	0.5	0.0	41.9	16.0	25.9
Pip	259.6	2.2	627	34	0.1	0.1	44.3	13.4	30.9
Pip	277.2	3.6	3423	21	0.3	0.2	41.6	14.8	26.8
Lev	364.4	3.8	553	18	0.5	0.3	53.4	12.5	41.1
Lev	350.7	3.8	708	14	0.5	0.1	49.8	13.2	36.6
Lev	356.4	3.5	600	20	0.3	0.2	49.8	12.2	40.2
PBS	224.8	3.0	782	25	0.2	0.2	48.3	15.6	32.7
PBS	298.0	2.3	1321	15	0.0	0.0	49.4	13.2	36.2
PBS	261.0	2.9	775	21	0.2	0.0	46.5	16.5	30.0

AST, aspartate aminotransferase; ALT, alanine transaminase; ALP, alkaline phosphatase; GT, glutamyl transferase; TB, total bilirubin; DB, direct bilirubin; TP, total protein; ALBU, albumin; Globu, globulins; PBS, phosphate-buffered saline; CAFe, *Capsicum annuum* L. ethanol extract; CAFa, *Capsicum annuum* L. acetone extract; CPLe, *Carica papaya* L. ethanol extract; CPLa, *Carica papaya* L. acetone extract; Pip, Piperazine citrate; Lev, Levamisole hydrochloride.

**TABLE 5:** Multiple comparisons of liver function parameters.

(I) Treatment	(J) Treatment	Mean ± s.d.	Mean difference (I–J)	Standard error	Sig.	95% confidence interval	
						Lower bound	Upper bound
**Dependent variable: Albumin**							
CAFe	CAFa	13.633 ± 0.72	−1.833	1.0060	0.555	−5.268	1.602
	CPLa	13.233 ± 1.38	−1.433	1.0060	0.781	−4.868	2.002
	CPLe	15.733 ± 0.71	−3.933*	1.0060	0.020*	−7.368	−0.498
	Lev	12.633 ± 0.51	−0.833	1.0060	0.978	−4.268	2.602
	PBS	15.100 ± 1.71	−3.300	1.0060	0.063	−6.735	0.135
	Pip	14.733 ± 1.30	−2.933	1.0060	0.119	−6.368	0.502
	CAFe	11.800 ± 1.68					
**Dependent variable: AST**							
Lev	CAFa	263.167 ± 21.4	94.000	29.2865	0.072	−6.001	194.001
	CAFe	253.667 ± 46.26	103.500*	29.2865	0.040*	3.499	203.501
	CPLa	276.333 ± 49.64	80.833	29.2865	0.153	−19.168	180.835
	CPLe	324.867 ± 49.79	32.300	29.2865	0.917	−67.701	132.301
	PBS	261.267 ± 36.60	95.900	29.2865	0.064	−4.101	195.901
	Pip	268.567 ± 8.81	88.600	29.2865	0.099	−11.401	188.601
	Lev	357.167 ± 6.88					

Note: There was a significant difference in the level of albumin in the chicken of CAFe and CPLe (*p* = 0.002). There was a significant difference in the level of AST in the chicken of CAFe and levamisole hydrochloride (*p* = 0.04).

s.d., standard deviation; Sig., significant; AST, aspartate aminotransferase; CAFe, *Capsicum annuum* L. ethanol extract; CAFa, *Capsicum annuum* L. acetone extract; CPLe, *Carica papaya* L. ethanol extract; CPLa, *Carica papaya* L. acetone extract; Pip, Piperazine citrate; Lev, Levamisole hydrochloride; PBS, Phosphate buffered saline.

* , *p* < 0.05.

There was no significant difference between the effects of the plant extracts and piperazine citrate on liver function parameters (*p* > 0.05). There was no significant difference between the effects of the plant extracts and levamisole hydrochloride on liver function parameters except for serum AST, where levamisole hydrochloride caused significantly higher levels of AST compared to CAFe (*p* = 0.04), see Table 5.

### Haematology

The results of the haematological parameters are shown in Table 6.

**TABLE 6:** Haematological parameters from chickens on herbal and synthetic anthelmintics.

Treatment	PCV (%)	TPP (g/dL)	FIB (g/dL)	TWBC (uL)	TRBC (uL)	HB (g/dL)	EOS (%)	MON (%)	LYM (%)	HTR (%)	BAND (%)
CAFa	29.5	2.80	0.20	7.90	2.70	7.5	3	8	50	42	4
CAFa	24.0	3.50	0.20	8.80	2.70	6.8	2	2	45	46	5
CAFa	25.0	3.30	0.30	10.50	4.40	7.4	1	4	24	71	3
CAFe	29.5	4.00	0.20	10.30	3.60	6.4	2	4	34	59	1
CAFe	27.0	4.20	0.10	6.40	3.90	6.0	4	5	34	52	10
CAFe	35.0	4.20	0.10	15.20	4.60	8.2	3	2	50	42	6
CPLa	22.0	3.00	0.10	5.00	2.60	5.0	4	4	44	40	4
CPLa	30.0	3.40	0.10	3.30	3.10	7.0	5	11	81	8	3
CPLa	25.0	3.10	0.10	8.90	2.80	7.0	6	9	60	34	6
CPLe	32.0	2.50	0.10	4.50	6.00	7.8	4	6	66	22	2
CPLe	31.0	4.20	0.10	3.00	3.60	10.0	8	8	48	44	3
CPLe	23.0	2.40	0.10	22.80	2.40	6.0	3	9	31	68	1
Pip	29.0	3.30	0.30	8.50	3.10	7.6	1	7	44	48	2
Pip	30.0	3.60	0.10	8.20	4.50	8.4	3	11	36	53	3
Pip	31.0	3.80	0.20	5.00	4.80	8.0	2	12	34	50	2
Lev	23.0	3.60	0.10	3.10	2.30	6.0	8	22	34	30	6
Lev	28.0	4.30	0.20	7.00	4.00	7.2	12	10	36	38	4
Lev	25.5	3.95	0.15	5.05	3.15	6.6	10	16	35	34	5
PBS	30.0	3.00	0.10	12.40	4.90	8.0	4	2	46	48	7
PBS	31.0	3.50	0.10	4.70	3.60	6.0	2	5	38	60	5
PBS	27.0	3.40	0.20	6.00	2.50	7.5	2	6	56	42	3

PCV, packed cell volume; TPP, total plasma proteins; FIB, fibrinogen; TWBC, total white blood cells; TRBC, total red blood cells; HB, haemoglobin; EOS, eosinophils; MON, monocytes; LYM, lymphocytes; HTR, heterophils. All the treatments were done in triplicates; PBS, phosphate-buffered saline; CAFe, *Capsicum annuum* L. ethanol extract; CAFa, *Capsicum annuum* L. acetone extract; CPLe, *Carica papaya* L. ethanol extract; CPLa, *Carica papaya* L. acetone extract; Pip, Piperazine citrate; Lev, Levamisole hydrochloride.

There were no significant differences in the haematological parameters when comparing chickens treated with CPLa, CAFa, CPLe, CAFe, piperazine citrate, levamisole hydrochloride and those in the control group, except for eosinophils. There was a significant difference in the percentage of eosinophil between chickens exposed to CPLa and the control group (*p* = 0.017). There was a significant difference in the percentage of eosinophils between chickens exposed to CAFa and the control group (*p* = 0.000). There was a significant difference in the percentage of eosinophils between chickens exposed to CPLe and the control group (*p* = 0.017). There was a significant difference in the percentage of eosinophils between chickens exposed to CAFe and the control group (*p* = 0.001). There was a significant difference in the percentage of eosinophils between chickens exposed to levamisole hydrochloride and the control group (*p* = 0.001). There was a significant difference in the percentage of eosinophils between chickens exposed to piperazine citrate and the control group (*p* = 0.000) (Table 7).

**TABLE 7:** Multiple comparisons of haematological parameters in chickens treated with herbal and synthetic anthelmintics.

(I) Treatment	Tukey HSD						
	(J) Treatment	Mean ± s.d.	Mean difference (I-J)	Std. error	Sig.	95% confidence interval	
						Lower bound	Upper bound
**Dependent variable: PCV %**							
Control	CPLa	25.667 ± 4.04	−0.1670	2.71830	1.000	−9.4480	9.1150
	CAFa	26.167 ± 2.93	−0.6670	2.71830	1.000	−9.9480	8.6150
	CPLe	28.667 ± 4.93	−3.1670	2.71830	0.896	−12.4480	6.1150
	CAFe	30.500 ± 4.09	−5.0000	2.71830	0.545	−14.2820	4.2820
	Lev	25.500 ± 2.50	−3.8330	2.71830	0.788	−13.1150	5.4480
	Pip	30.000 ± 1.00	−4.5000	2.71830	0.653	−13.7820	4.7820
	Control	29.333 ± 2.08					
**Dependent variable: TPP g/dL**							
Control	CPLa	3.167 ± 0.21	0.7833	0.37364	0.404	−0.4925	2.0591
	CAFa	3.200 ± 0.36	0.7500	0.37364	0.451	−0.5258	2.0258
	CPLe	3.033 ± 1.01	0.9167	0.37364	0.247	−0.3591	2.1925
	CAFe	4.133 ± 0.11	−0.1833	0.37364	0.999	−1.4591	1.0925
	Lev	3.950 ± 0.35	0.6500	0.37364	0.604	−0.6258	1.9258
	Pip	3.567 ± 0.25	0.3833	0.37364	0.939	−0.8925	1.6591
	Control	3.300 ± 0.26					
**Dependent variable: Fib g/dL**							
Control	CPLa	0.100 ± 0.00	0.0500	0.04629	0.924	−0.1081	0.2081
	CAFa	0.233 ± 0.06	−0.0833	0.04629	0.568	−0.2414	0.0747
	CPLe	0.100 ± 0.00	0.0500	0.04629	0.924	−0.1081	0.2081
	CAFe	0.133 ± 0.06	0.0167	0.04629	1.000	−0.1414	0.1747
	Lev	0.150 ± 0.05	0.0167	0.04629	1.000	−0.1414	0.1747
	Pip	0.200 ± 0.10	−0.0500	0.04629	0.924	−0.2081	0.1081
	Control	0.133 ± 0.06					
**Dependent variable: TWBC/uL**							
Control	CPLa	5.733 ± 2.87	−0.6833	4.08850	1.000	−14.6439	13.2772
	CAFa	9.067 ± 1.32	−4.0167	4.08850	0.950	−17.9772	9.9439
	CPLe	10.100 ± 11.02	−5.0500	4.08850	0.869	−19.0105	8.9105
	CAFe	10.633 ± 4.41	−5.5833	4.08850	0.810	−19.5439	8.3772
	Lev	5.050 ± 1.95	−2.6500	4.08850	0.994	−16.6105	11.3105
	Pip	7.233 ± 1.94	−2.1833	4.08850	0.998	−16.1439	11.7772
	Control	7.700 ± 4.12					
**Dependent variable: TRBC/uL**							
Control	CPLa	2.833 ± 0.25	0.3167	0.85296	1.000	−2.5958	3.2292
	CAFa	3.267 ± 0.98	−0.1167	0.85296	1.000	−3.0292	2.7958
	CPLe	4.000 ± 1.83	−0.8500	0.85296	0.947	−3.7625	2.0625
	CAFe	4.033 ± 0.51	−0.8833	0.85296	0.937	−3.7958	2.0292
	Lev	3.150 ± 0.85	−0.5167	0.85296	0.996	−3.4292	2.3958
	Pip	4.133 ± 0.91	−0.9833	0.85296	0.900	−3.8958	1.9292
	Control	3.667 ± 1.20					
**Dependent variable: HB g/dL**							
Control	CPLa	6.333 ± 1.55	0.2670	0.89800	1.000	−2.8000	3.3330
	CAFa	7.233 ± 0.38	−0.6330	0.89800	0.990	−3.7000	2.4330
	CPLe	7.933 ± 2.01	−1.3330	0.89800	0.749	−4.4000	1.7330
	CAFe	6.667 ± 1.17	−0.2670	0.89800	1.000	−3.3330	2.8000
	Lev	6.600 ± 0.60	−0.5670	0.89800	0.994	−3.6330	2.5000
	Pip	8.00 ± 0.40	−1.4000	0.89800	0.708	−4.4660	1.6660
	Control	7.167 ± 1.04					
**Dependent variable: Eos %**							
Control	CPLa	5.000 ± 1.00	5.0000*	1.24700	0.017*	0.7400	9.2600
	CAFa	2.000 ± 1.00	8.0000*	1.24700	0.000**	3.7400	12.2600
	CPLe	5.000 ± 2.65	5.0000*	1.24700	0.017*	0.7400	9.2600
	CAFe	3.000 ± 1.00	7.0000*	1.24700	0.001**	2.7400	11.2600
	Lev	10.000 ± 2.00	7.3300*	1.24700	0.001**	3.0700	11.5900
	Pip	2.000 ± 1.00	8.0000*	1.24700	0.000**	3.7400	12.2600
	Control	2.670 ± 1.16					
**Dependent variable: Mon %**							
Control	CPLa	8.00 ± 3.61	−3.6700	2.66100	0.804	−12.7500	5.4200
	CAFa	4.67 ± 3.06	−0.3300	2.66100	1.000	−9.4200	8.7500
	CPLe	7.67 ± 1.53	−3.3300	2.66100	0.862	−12.4200	5.7500
	CAFe	3.67 ± 1.53	0.6700	2.66100	1.000	−8.4200	9.7500
	Lev	16.00 ± 6.00	−11.6700*	2.66100	0.009**	−20.7500	−2.5800
	Pip	10.00 ± 2.65	−5.6700	2.66100	0.388	−14.7500	3.4200
	Control	4.33 ± 2.08					
**Dependent variable: LMP %**							
Control	CPLa	61.670 ± 18.56	−26.6700	9.93600	0.173	−60.6000	7.2600
	CAFa	39.670 ± 13.80	−4.6700	9.93600	0.999	−38.6000	29.2600
	CPLe	48.330 ± 17.50	−13.3300	9.93600	0.822	−47.2600	20.6000
	CAFe	39.330 ± 9.24	−4.3300	9.93600	0.999	−38.2600	29.6000
	Lev	35.000 ± 1.00	−11.6700	9.93600	0.893	−45.6000	22.2600
	Pip	38.000 ± 5.29	−3.0000	9.93600	1.000	−36.9300	30.9300
	Control	46.67 ± 9.02					
**Dependent variable: HTR %**							
Control	CPLa	27.330 ± 17.01	6.6700	10.88900	0.995	−30.5100	43.8500
	CAFa	53.000 ± 15.72	−19.0000	10.88900	0.601	−56.1800	18.1800
	CPLe	44.670 ± 23.01	−10.6700	10.88900	0.951	−47.8500	26.5100
	CAFe	51.000 ± 8.54	−17.0000	10.88900	0.707	−54.1800	20.1800
	Lev	34.000 ± 4.00	−16.0000	10.88900	0.757	−53.1800	21.1800
	Pip	50.330 ± 2.52	−16.3300	10.88900	0.741	−53.5100	20.8500
	Control	50.000 ± 9.17					
**Dependent variable: Band %**							
Control	CPLa	4.330 ± 1.53	0.6700	1.69000	1.000	−5.1100	6.4400
	CAFa	4.000 ± 1.00	1.0000	1.69000	0.996	−4.7700	6.7700
	CPLe	2.000 ± 1.00	3.0000	1.69000	0.583	−2.7700	8.7700
	CAFe	5.670 ± 4.51	−0.6700	1.69000	1.000	−6.4400	5.1100
	Lev	5.000 ± 1.00	0.0000	1.69000	1.000	−5.7700	5.7700
	Pip	2.33 ± 0.50	2.6700	1.69000	0.697	−3.1100	8.4400
	Control	5.000 ± 2.00					

Note: There was no significant difference between the treatments and control (PBS) regarding PCV, TPP, Fib, TWBC, TRBC, Hb, LMP, HTR and Band. The number of eosinophils was significantly different between CPLa and control (*p* = 0.017), CAFa and control (*p* = 0.000), CPLe and control (*p* = 0.017), CAFe and control (*p* = 0.01), levamisole hydrochloride and control (*p* = 0.001) and piperazine citrate and control (*p* = 0.000). The number of monocytes was significantly different between levamisole hydrochloride and control (*p* = 0.009).

HTR, heterophils; PCV, packed cell volume; TPP, total plasma proteins; FIB, fibrinogen; TWBC, total white blood cells; TRBC, total red blood cells; HB, haemoglobin; EOS, eosinophils; MON, monocytes; LMP, lymphocytes; s.d., standard deviation; Std, standard; Sig., significant; CAFe, *Capsicum annuum* L. ethanol extract; CAFa, *Capsicum annuum* L. acetone extract; CPLe, *Carica papaya* L. ethanol extract; CPLa, *Carica papaya* L. acetone extract; Pip, Piperazine citrate; Lev, Levamisole hydrochloride; PBS, Phosphate buffered saline.

* , *p* < 0.05;

** , *p* < 0.01.

There was no significant difference in haematological parameters when comparing chickens that were treated with plant extracts (CPLa, CPLe, CAFa and CAFe) and those that were treated with synthetic anthelmintics (piperazine citrate and levamisole hydrochloride) (*p* > 0.05).

### Organ histopathology

#### Heart

**FIGURE 1:**
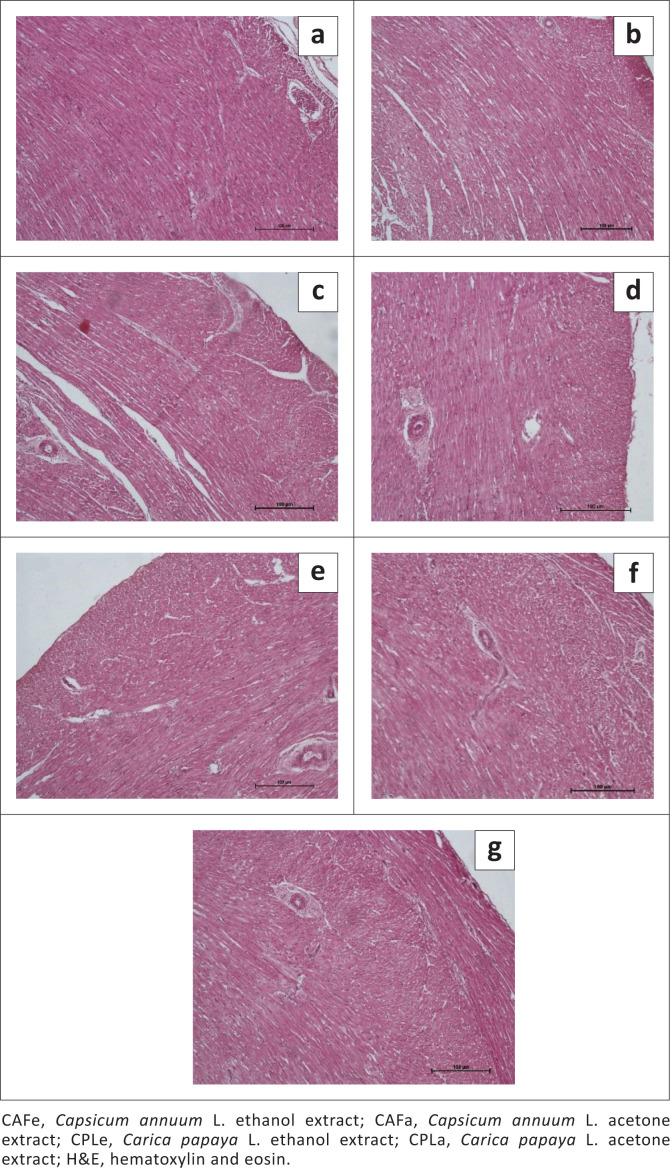
Histopathology micrographs of heart sections from chickens treated with plant extracts and synthetic anthelmintics (H&E stain): (a) heart of chicken treated with CAFa, (b) heart of chicken treated with CAFe, (c) heart of chicken treated with CPLa, (d) heart of chicken treated with CPLe, (e) heart of chicken treated with piperazine, (f) heart of chicken treated with levamisole and (g) heart of chicken treated with PBS (control). In all treatments, there were no lesions in the heart.

For all treatments, there was no necrosis of myocardium, no foci of haemorrhages or form of vascular damage observed, Figure 1.

#### Kidneys

**FIGURE 2:**
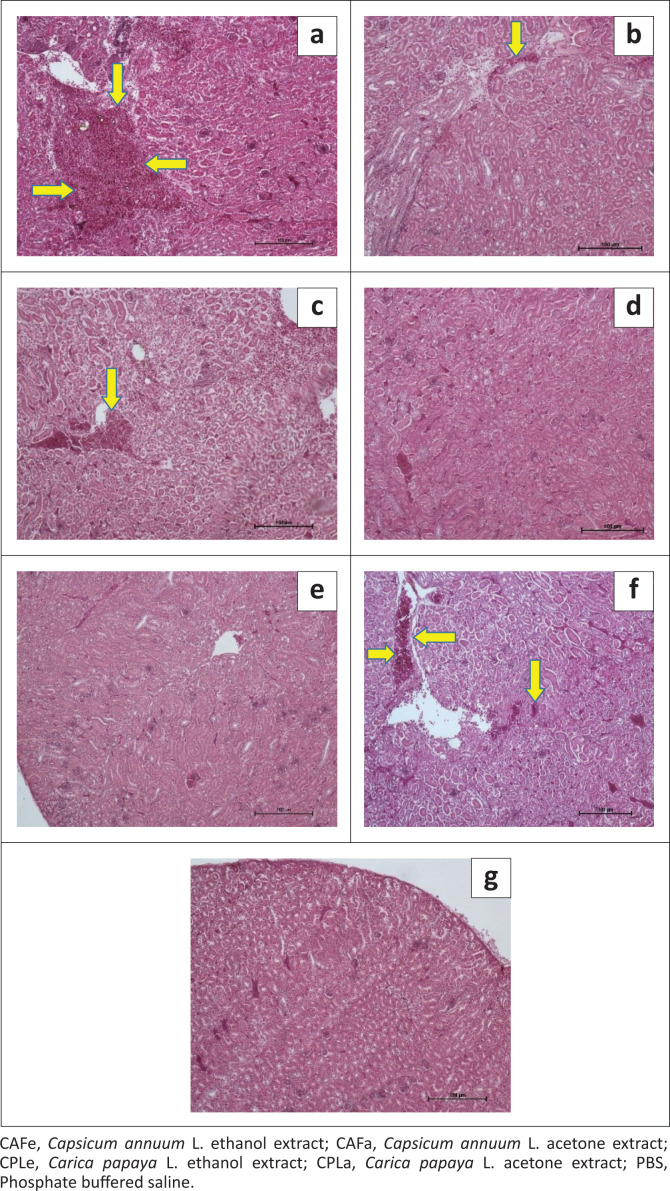
Histopathology micrographs of kidney sections from chickens treated with plant extracts and synthetic anthelmintics: (a) kidney of chicken treated with CAFa (wide haemorrhages in renal cortex and medulla), (b) kidney of chicken treated with CAFe (renal haemorrhages and interstitial nephritis), (c) kidney of chicken treated with CPLa (renal congestion and haemorrhages), (d) kidney of chicken treated with CPLe (no observable lesions), (e) kidney of chicken treated with piperazine (no observable lesions), (f) kidney of chicken treated with levamisole (renal congestion and haemorrhages), (g) kidney of chicken treated with PBS (no observable lesions).

CAFa caused observable kidney lesions in 1 out of 3 of the chickens, CAFe caused observable kidney lesions in all the three chickens (*n* = 3/3), CPLa caused observable kidney lesions (*n* = 2/3) chickens, CPLe did not cause any observable lesions in any of the three chickens (*n* = 0/3), piperazine citrate did not cause any observable lesions in any of the three chickens (*n* = 0/3), levamisole hydrochloride caused observable kidney lesions in 1 out of 3 of the chickens, PBS did not cause any observable lesions in any of the three chickens (*n* = 0/3), Figure 2.

#### Liver

**FIGURE 3:**
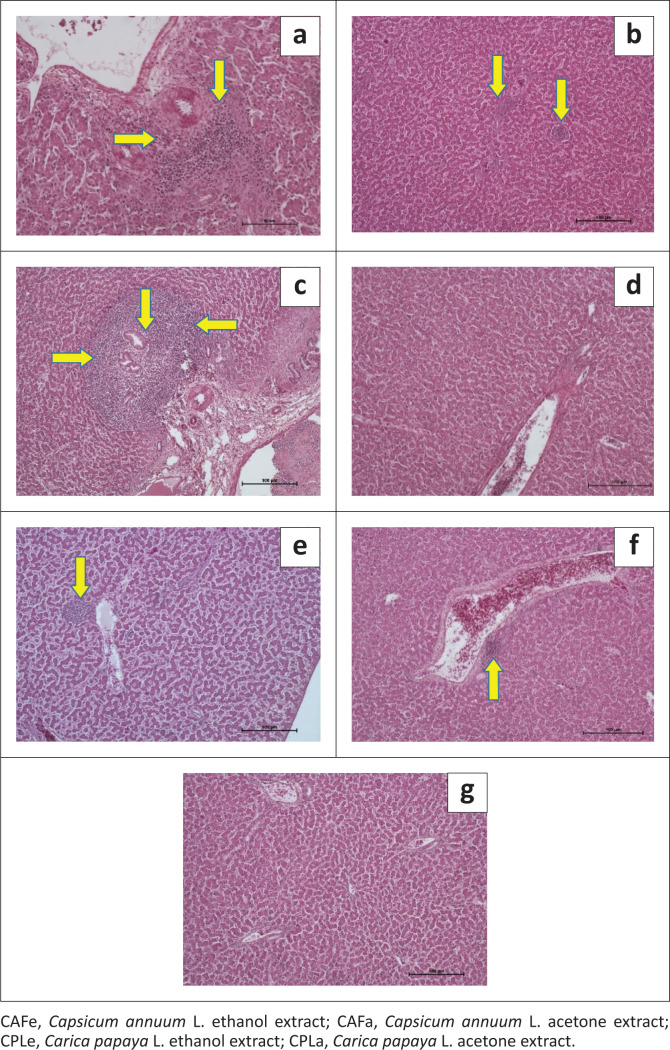
Histopathology micrograph of liver sections from chickens treated with plant extracts and synthetic anthelmintics: (a) liver of chicken treated with CAFa (severe periportal necrotising hepatitis), (b) liver of chicken treated with CAFe (multi-focal hepatitis), (c) liver of chicken treated with CPLa (severe widespread periportal necrotising hepatitis), (d) liver of chicken treated with CPLe (no observable lesions), (e) liver of chicken treated with piperazine (periportal hepatitis), (f) liver of chicken treated with levamisole (moderate periportal hepatitis) and (g) liver of chicken treated with PBS (no observable lesions).

CAFa caused observable liver lesions in 1 out of 3 chickens, CAFe caused observable liver lesions in 1 out of 3 chickens, CPLa caused observable liver lesions in 1 out of 3 chickens, CPLe did not cause any observable liver lesions in any of the three chickens (*n* = 0/3), PBS did not cause any observable liver lesions in any of the three chickens (*n* = 0/3), piperazine citrate caused observable liver lesions in 1 out of 3 chickens and levamisole hydrochloride caused observable liver lesions in 1 out of 3 chickens, Figure 3.

### Summary of organ toxicity findings

The safety score was assumed as: safe < 1 out of 9 > not safe. The order of toxicity was CPLe and control (*n* = 0/9), piperazine citrate (*n* = 1/9), levamisole hydrochloride and CAFa (*n* = 2/9), CPLa (*n* = 3/9) and CAFe (*n* = 4/9).

*CAFa* caused no observable lesions in chicken hearts. It caused observable lesions in the kidneys of 1 out of 3 chickens and in the liver of 1 out of 3 chickens. It generally caused a toxicity score of two out of nine (*n* = 2/9) and was considered not safe.

*CAFe* caused no observable lesions in chicken hearts. It caused observable lesions in the kidneys of 3 out of 3 chickens and in the liver of 1 out of 3 chickens. Its general toxicity score was four out of nine (*n* = 4/9) and was therefore considered not safe.

*CPLa* caused no observable lesions in chicken hearts. It caused observable lesions in the kidneys of two chickens out of three (*n* = 2/3) and in the liver of one chicken out of three chickens (*n* = 1/3). Its total toxicity score was three out of nine (*n* = 3/9) and was also considered not safe.

*CPLe* caused no observable lesions in chicken hearts, kidneys or liver. Its total toxicity score was 0 out of 9 (*n* = 0/9), the same as *PBS* and was considered safe.

*Piperazine citrate* caused no observable lesions in the chicken hearts or kidneys but caused observable lesions in the liver of 1 out of 3 chickens (*n* = 1/3). Its total toxicity score was 1 out of 9 (*n* = 1/9) and was considered moderately safe.

*Levamisole hydrochloride* caused no observable lesions in the chicken hearts but caused observable lesions in the kidneys of 1 out of 3 chickens (*n* = 1/3) and in the liver of 1 out of 3 chickens (*n* = 1/3). Its total toxicity score was 2 out of 9 (*n* = 2/9) and was considered not safe.

## Discussion

The results were analysed by linear regressions followed by Tukey’s honestly significant difference test. CAFa, CAFe, CPLa and CPLe, when compared to PBS, did not significantly affect renal function parameters; however, CAFa needs to be used with caution because it led to lower sodium in blood. The extracts did not show significant effects on the electrolyte balance with regard to BUN, sodium, chloride, creatinine and uric acid. Reading renal functions, the extracts are safe for use at the given concentrations. Although the extracts did not affect the sodium electrolyte balance, CAFa caused minor lowering of blood sodium compared to other extracts and piperazine citrate. Lower blood sodium predisposes to decreased blood pressure, increased uric acid, increased haematocrit, shock and heart failure (Julian 1987; Sad 2016). The plant extracts were as safe as the synthetic anthelmintics regarding their effects on BUN, sodium, chloride, creatinine and uric acid balance. The extracts, as well as piperazine citrate and levamisole hydrochloride, had no effects on the metabolic actions that yield uric acid. The treatments did not affect the glucose transporter 9, which regulates serum uric acid in chicken (Ding et al. 2021). The extracts did not significantly affect the liver functions. Chronic toxicity studies are required to confirm the findings. However, CPLe caused higher albumin compared to CAFe, implying that it led to greater dehydration than CAFe. Levamisole hydrochloride increased AST compared to CAFe, implying that levamisole chloride is more likely to cause liver damage compared to CAFe (Khanam et al. 2016).

The plant extracts, as well as piperazine and levamisole, caused increased blood eosinophils. Although helminth infestations can also cause eosinophilia (Fuentebella, Fridge & Bass 2011; Maxwell 1987), the findings point to more than that. The placebo with higher helminth loads had less blood eosinophils compared to the treatments possibly because the inflammatory action of the treatments was greater than that of the helminths, which leads to eosinophilia. Eosinophils are involved in the starting inflammatory responses, can act as antigen-presenting cells, are particularly responsible for defence against parasitic infections and modulate host’s general immune responses (Rothenberg & Hogan 2006). Like piperazine and levamisole, the extracts possibly induced hypersensitivity, autoimmune disorders or neoplasm defence mechanisms, which manifest as eosinophilia (Kanuru & Sapra 2023); however, the mechanisms driving eosinophilia require further investigation. The monocytosis observed in levamisole hydrochloride treatments implies that it caused more stress and autoimmune disorders compared to the control. Monocytosis is observed in stressful conditions and during the onset of autoimmune disorders (Taebipour et al. 2017). The plant extracts were as safe as piperazine citrate but safer than levamisole chloride regarding their effects on the haematological parameters balance.

The different plant extracts, piperazine and levamisole did not show any observable lesions in the heart muscle sections of chickens. The anthelmintic extracts are not toxic to the heart muscles and major vessels. The findings relating to *C. annuum* L. (acetone red pepper extract and ethanol red pepper extract) are in agreement with Mandal et al. (2023), who reported that *C. annuum* L. is cardio-protective. Sanati, Razavi and Hosseinzadeh (2017) also reported that *C. annuum* L. has beneficial effects on the heart. The findings relating to *C. papaya* L. (acetone pawpaw leaves extract and ethanol pawpaw leaves extract) are in agreement with Haramaki et al. (1995), Hasimun et al. (2020), Kong et al. (2021), who described the cardio-protective action of *C. papaya* L.

Acetone red pepper extract (CAFa), ethanol red pepper extract (CAFe) and acetone pawpaw leaves extract (CPLa) were toxic to kidneys (Figure 2). The actions of *C. annuum* L. are in agreement with Jabar and Jassim (2023), who observed kidney histological alterations among animals treated with *C. annuum* L. Yuca (2022) also reported kidney damages among animals that received very high doses of *C. annuum* L. The mechanisms of CPLa toxicity are unknown because *C. papaya* L. is reported in the literature as nephro-protective (Francis et al. 2020). It is possible that acetone yields some compounds with nephro-toxic effects compared to ethanol. However, further investigations are needed to prove the variations in the compounds yielded with the different solvents and the renal effects of such compounds. Levamisole hydrochloride was equally toxic to the kidneys, but piperazine citrate was safe. Nephro-toxic effects of levamisole have been reported in mice (Almawla & Al Baggou 2023); however, there are no such earlier reports in chickens. There is a need to confirm the findings regarding the safety of levamisole in chickens at the efficacious doses.

All extracts were toxic to the liver except CPLe; equally, piperazine and levamisole were toxic. The observed hepatotoxic effects of *C. annuum* L. in chickens are in disagreement with (Das et al. 2018; Effendi & Sukmanadi 2021; Oloruntola et al. 2024), who reported its hepato-protective actions in other species. The concentrations in this experiment were very high compared to the concentrations in literature where *C. annuum* L. is reported to have had protective action. *Carica papaya* L. is also reported to be hepato-protective as for CPLe (Awodele et al. 2016; Shaban et al. 2021). However, CPLa was toxic, possibly because of the difference in compound yields of acetone compared to ethanol. The hepatic findings of the effects of piperazine were in agreement with those of Bakhrebah, Abo-Znada and Ramadan (2011), who observed histological changes in the chicken liver. The findings regarding levamisole were in agreement with Miah et al. (2020), who reports that high doses of levamisole are detrimental to the liver. Overall, all plant extracts were toxic, except CPLe. Levamisole was also toxic, and piperazine was moderately toxic. CPLe was as safe as the placebo and safer than the two synthetic anthelmintics (piperazine and levamisole) that are commonly used by commercial chicken farmers in Uganda.

### Strength and limitations

The experimental design used the same cohort of chickens to triangulate the toxicity evaluation using the parameters of haematology, renal function tests, liver function tests and organ histotoxicity. This integrated assessment is used to derive a trustable conclusion. The concentrations of the extracts used were proven in previous efficacy tests. The concentration of piperazine citrate and levamisole hydrochloride was that recommended by the manufacturers and other researchers. Phosphate-buffered saline treatment is included as the placebo.

The study focusses on the toxic effects at the effective concentration of the extracts. The effects of extremely high doses were not studied. Immediate effects were not studied because samples were collected 1 week after the last treatment. The study does not consider the effects of chronic use of the treatments because chickens were not kept for a year or 2 years. Isolated or individual parasite effects as possible confounders of toxicity were not assessed.

### Implications or recommendations

*Capsicum annuum* L. and *Carica papaya* L. are possible treatment alternatives to modern pharmaceuticals for chicken helminth infestation. The organ toxicity study showed that CPLe was the safest extract. Other extracts caused observable organ toxicity lesions in the order of CAFa < CPLa < CAFe. The study demonstrates the relative toxicity of piperazine citrate and levamisole hydrochloride at recommended concentrations compared to plant extracts used as anthelmintics in chickens.

We recommend evaluating the toxicity of pure extracts such that the most effective fractions are scaled up as alternatives to synthetic anthelmintics. Toxicological assessment after prolonged use of the extracts should be evaluated with a larger sample size before recommending for wider use in chickens. The study should be repeated in different breeds of chicken in various geographical zones to identify any possible variations. The safety concerns of piperazine citrate and levamisole hydrochloride are critical requiring more studies for confirmation and to make decisions on concentrations. Safety studies of highly purified extracts are recommended.

## Conclusion

The extracts *C. papaya* L. and *C. annuum* L. caused minor lowering of blood sodium and eosinophilia, and they were toxic to the kidneys and liver. Generally, there was no observed difference in safety between the plant anthelmintics and the synthetic anthelmintics (piperazine citrate and levamisole hydrochloride); all should be used with caution.

## References

[CIT0001] Abdel-Daim, M.M., Abo-EL-Sooud, K., Aleya, L., Bungau, S.G., Najda, A. & Saluja, R., 2018, ‘Alleviation of drugs and chemicals toxicity: Biomedical value of antioxidants’, *Oxidative Medicine and Cellular Longevity* 2018, 3–5. 10.1155/2018/6276438 PMC6311870 30647814

[CIT0002] Almawla, F.F. & Al Baggou, B.K., 2023, ‘Acute toxic effects of levamisole and ivermectin in mice’, *Journal of Applied Veterinary Sciences* 8(3), 75–81. 10.21608/JAVS.2023.213855.1236

[CIT0003] Awodele, O., Yemitan, O., Ise, P.U. & Ikumawoyi, V.O., 2016, ‘Modulatory potentials of aqueous leaf and unripe fruit extracts of Carica papaya Linn. (Caricaceae) against carbon tetrachloride and acetaminophen-induced hepatotoxicity in rats’, *Journal of Intercultural Ethnopharmacology* 5(1), 27–35. 10.5455/jice.20160124113528 27069723 PMC4805144

[CIT0004] Bakhrebah, A.O., Abo-Znada, N.Y. & Ramadan, H., 2011, ‘The efficacy of garlic (Allium sativum) in comparison with the anthelmintic piperazine adipate in the recovery of the tissue damage caused by Ascaridia galli infection in experimentally infected chickens’, in Proceedings of the 3rd scientific conference of animal wealth research in the middle East and North Africa. Foreign Agricultural Relations (FAR), Egypt, 29th November–1 December 2010, pp. 576–582, Cairo.

[CIT0005] Baudoux, T. & Nortier, J.L., 2017, ‘Nephrotoxicity of Herbal Products’, in O. Pelkonen, P. Duez, P. Vuorela & H. Vuorela (eds.), *Toxicology of Herbal Products*, Springer, Cham.

[CIT0006] Boyal, R.S., Buhr, R.J., Harris, C.E., Jacobs, L. & Bourassa, D.V., 2020, ‘Equipment and methods for poultry euthanasia by a single operator’, *Journal of Applied Poultry Research* 29(4), 1020–1032. 10.1016/J.JAPR.2020.09.010

[CIT0007] Cojean, O., Larrat, S. & Vergneau-Grosset, C., 2020, ‘Clinical management of avian renal disease’, *Veterinary Clinics of North America – Exotic Animal Practice* 23(1), 75–101. 10.1016/j.cvex.2019.08.004 31759453 PMC7129257

[CIT0008] Comar, S.R., Malvezzi, M. & Pasquini, R., 2017, ‘Evaluation of criteria of manual blood smear review following automated complete blood counts in a large university hospital’, *Revista Brasileira de Hematologia e Hemoterapia* 39(4), 306–317. 10.1016/j.bjhh.2017.06.007 29150102 PMC5693276

[CIT0009] Das, M., Basu, S., Banerjee, B., Sen, A., Jana, K. & Datta, G., 2018, ‘Hepatoprotective effects of green Capsicum annum against ethanol induced oxidative stress, inflammation and apoptosis in rats’, *Journal of Ethnopharmacology* 227, 69–81. 10.1016/j.jep.2018.08.019 30118838

[CIT0010] Dey, P., 2018, ‘Processing of tissue in histopathology laboratory’, in P. Dey (ed.), *Basic and advanced laboratory techniques in histopathology and cytology*, pp. 19–27, Springer Singapore, Singapore.

[CIT0011] Ding, X., Peng, C., Li, S., Li, M., Li, X., Wang, Z. et al., 2021, ‘Chicken serum uric acid level is regulated by glucose transporter 9’, *Animal Bioscience* 34(4), 670–679. 10.5713/ajas.20.0092 32810934 PMC7961270

[CIT0012] Effendi, M.H. & Sukmanadi, M., 2021, ‘Analysis of Capsicum annuum L. Methanolic extract and Its potential as a hepatoprotector’, *Indian Journal of Forensic Medicine & Toxicology* 15(3), 3679–3684. 10.37506/ijfmt.v15i3.15869

[CIT0013] El-Kholy, H. & Kemppainen, B.W., 2005, ‘Levamisole residues in chicken tissues and eggs’, *Poultry Science* 84(1), 9–13. 10.1093/PS/84.1.9 15685936

[CIT0014] Francis, Y.M., Vijayakumar, J., Raghunath, G., Vijayalakshmi, S. & Sivanesan, S., 2020, ‘Protective effect of Carica papaya leaf extract against mercuric chloride – Induced nephrotoxicity in Wistar rats’, *Pharmacognosy Magazine* 16(70), 379–384. 10.4103/pm.pm_11_20

[CIT0015] Fuentebella, J., Fridge, J.L. & Bass, D.M., 2011, ‘Enteric parasites’, *Pediatric Gastrointestinal and Liver Disease* 2011, 423–434.e4. 10.1016/B978-1-4377-0774-8.10040-5

[CIT0016] González-Miqueo, L., Elustondo, D., Lasheras, E., Bermejo, R. & Santamaría, J.M., 2010, ‘Heavy metal and nitrogen monitoring using moss and topsoil samples in a Pyrenean forest catchment’, *Water, Air, and Soil Pollution* 210(1–4), 335–346. 10.1007/s11270-009-0256-9

[CIT0017] Gounden, V., Bhatt, H. & Jialal, I., 2021, *Renal function tests*, StatPearls, viewed 13 April 2021, from http://www.ncbi.nlm.nih.gov/pubmed/29939598. 29939598

[CIT0018] Habeeb, S., 2010, ‘Ethno-veterinary and medical knowledge of crude plant extracts and its methods of application (traditional and modern) for tick control’, *World Applied Sciences Journal* 11(9), 1047–1054.

[CIT0019] Haramaki, N., Marcocci, L., Anna, R.D., Yan, L., Kobuchi, H., Packer, L. et al., 1995, ‘Fermented papaya preparation supplementation: Effect on oxidative stress to isolated rat hearts’, *Biochemistry and Molecular Biology International* 36(6), 1263–1268. 8535298

[CIT0020] Hasimun, P., Sulaeman, A., Dwi, I. & Maharani, P., 2020, ‘Supplementation of Carica papaya leaves (Carica papaya L.) in Nori preparation reduced blood pressure and arterial stiffness on hypertensive animal model’, *Journal of Young Pharmacists* 12(1), 63–66. 10.5530/jyp.2020.12.12

[CIT0021] Hirondart, M., Rombaut, N., Fabiano-Tixier, A.S., Bily, A. & Chemat, F., 2020, ‘Comparison between pressurized liquid extraction and conventional Soxhlet extraction for rosemary antioxidants, yield, composition, and environmental footprint’, *Foods* 9(5), 584. 10.3390/FOODS9050584 32380668 PMC7278715

[CIT0022] Jabar, Z.S. & Jassim, B.A., 2023, ‘Histological study of the Chilli Pepper (Capsicum annuum L.) extract on adipose tissue in White Mice (Mus musculus)’, *Annals of Agri Bio Research* 28(2), 367–371.

[CIT0023] Julian, R.J., 1987, ‘The effect of increased sodium in the drinking water on right ventricular hypertrophy, right ventricular failure and ascites in broiler chickens’, *Avian Pathology* 16(1), 61–71. 10.1080/03079458708436353 18766592

[CIT0024] Kanuru, S. & Sapra, A., ‘Eosinophilia’, in *StatPearls [Internet]*, U.S. National Library of Medicine, viewed 21 June 2023, from www.ncbi.nlm.nih.gov/books/NBK560929/.

[CIT0025] Kasozi, K.I., Otim, E.O., Ninsiima, H.I., Zirintunda, G., Tamale, A., Ekou, J. et al., 2021, ‘An analysis of heavy metals contamination and estimating the daily intakes of vegetables from Uganda’, *Toxicology Research and Application* 5, 239784732098525. 10.1177/2397847320985255

[CIT0026] Ke, C.H., Chen, J.W. & Lin, C.S., 2024, ‘Surveillance of drug residue profiles in Gallus gallus domesticus (Silkie Chickens) in Taiwan’, *Animals* 14(23), 3529. 10.3390/ani14233529 39682494 PMC11639894

[CIT0027] Kelly, L. & Alworth, L., 2013, ‘Techniques for collecting blood from the domestic chicken’, *Lab Anim al* 42, 359–361. 10.1038/laban.394 24051638

[CIT0028] Khanam, F., Iqbal, M.N., Ashraf, A., Yunus, F.-ul-N., Alam, S., Muhammad, A. et al., 2016, ‘Evaluation of changes in liver enzymes in broiler chicks (Gallous domesticus)’, *PSM Veterinary Research* 1(1), 26–31, viewed n.d., from https://psmjournals.org/index.php/vetres/article/view/82

[CIT0029] Kluwe, W.M., 1981, ‘Renal function tests as indicators of kidney injury in subacute toxicity studies’, *Toxicology and Applied Pharmacology* 57(3), 414–424. 10.1016/0041-008X(81)90239-8 7222048

[CIT0030] Kong, Y.R., Jong, Y.X., Balakrishnan, M., Bok, Z.K., Kwan, J., Weng, K. et al., 2021, ‘Beneficial role of Carica papaya extracts and phytochemicals on oxidative stress and related diseases: A mini review’, *Biology* 10(4), 1–20. 10.3390/biology10040287 PMC8066973 33916114

[CIT0031] Kuropka, P., Leśków, A., Małolepsza-Jarmołowska, K., Dobrzyński, M., Tarnowska, M., Majda, J. et al., 2022, ‘Effect of a single and triple dose of levamisole on hematological parameters in controlled inflammation model’, *Animals* 12(16), 1–11. 10.3390/ani12162110 PMC9404755 36009703

[CIT0032] Latif, N., Cigarroa, N., Furman, M., Feher, A., Maayah, M., Khokhar, A. et al., 2024, ‘Association of autoimmune disease with coronary vasomotor disorders in patients with angina and no obstructive coronary artery disease’, *Journal of the American College of Cardiology* 83(13), 1324. 10.1016/S0735-1097(24)03314-X38569762

[CIT0033] Mandal, S.K., Rath, S.K., Logesh, R., Mishra, S.K., Devkota, H.P. & Das, N., 2023, ‘Capsicum annuum L. and its bioactive constituents: A critical review of a traditional culinary spice in terms of its modern pharmacological potentials with toxicological issues’, *Phytotherapy Research* 37(3), 965–1002. 10.1002/ptr.7660 36255140

[CIT0034] Maxwell, M.H., 1987, ‘The avian eosinophil—A review’, *World’s Poultry Science Journal* 43(3), 190–207. 10.1079/WPS19870013

[CIT0035] McGaw, L.J. & Eloff, J.N., 2008, ‘Ethnoveterinary use of southern African plants and scientific evaluation of their medicinal properties’, *Journal of Ethnopharmacology* 119(3), 559–574. 10.1016/J.JEP.2008.06.013 18620038

[CIT0036] Miah, M., Shovon, A., Uddin, M., Hridoy, A. & Islam, M., 2020, ‘The effect of levamisole on growth performance, humoral immunity and blood biochemical profile in broiler chickens’, *Journal of Bangladesh Agricultural University* 18(S1), 858–863. 10.5455/jbau.13301

[CIT0037] National Research Council, 2006, *“Six New Approaches.” Toxicity testing for assessment of environmental agents: Interim report*, The National Academies Press, Washington, DC.

[CIT0038] OECD, 1996, *OECD guidelines for the testing of chemicals, test no.423: Acute oral toxicity class method*, OECD, Paris.

[CIT0039] Oloruntola, O.D., Ayodele, S.O., Oloruntola, D.A., Olarotimi, O.J., Falowo, A.B., Akinduro, V.O. et al., 2024, ‘Dietary supplementation of Capsicum powder affects the growth, immunoglobulins, pro-inflammatory cytokines, adipokines, meat, and liver histology of aflatoxin B1 exposed broiler chickens’, *Toxicon* 240, 107640. 10.1016/j.toxicon.2024.107640 38325757

[CIT0040] Rothenberg, M.E. & Hogan, S.P., 2006, ‘The eosinophil’, *Annual Review of Immunology* 24, 147–174. 10.1146/annurev.immunol.24.021605.090720 16551246

[CIT0041] Sad, N., 2016, ‘Electrolytes – Sodium, potassium and chlorides in poultry nutrition’, *Archives of Veterinary Medicine* 9(1), 31–42. 10.46784/e-avm.v9i1.95

[CIT0042] Sanati, S., Razavi, B.M. & Hosseinzadeh, H., 2017, ‘A review of the effects of Capsicum annuum L. and its constituent, capsaicin, in metabolic syndrome’, *Iranian Journal of Basic Medical Sciences*, 21(5), 439–448. 10.22038/IJBMS.2018.25200.6238 PMC6000222 29922422

[CIT0043] Sathiyanarayanan, L. & Arulmozhi, S., 2007, ‘Mucuna pruriens Linn – a comprehensive review’, *Pharmacognosy Review* 1(1), 157–162.

[CIT0044] Shaban, N.Z., El-Kot, S.M., Awad, O.M., Hafez, A.M. & Fouad, G.M., 2021, ‘The antioxidant and anti-inflammatory effects of Carica Papaya Linn. seeds extract on CCl 4-Induced liver injury in male rats. *BMC Complementary Medicine and Therapies* 9, 1–15. 10.1186/s12906-021-03479-9 PMC8719404 34969385

[CIT0045] Shumard, R.F., 1956, ‘The anthelmintic activity of powder and liquid parvex against *Ascaridia galli* and *Heterakis gallinae*’, *Journal of Parasitic Diseases* 42(2), 13.

[CIT0046] Ssempijja, F., Iceland Kasozi, K., Daniel Eze, E., Tamale, A., Ewuzie, S.A., Matama, K. et al., 2020, ‘Consumption of raw herbal medicines is associated with major public health risks amongst Ugandans’, *Journal of Environmental and Public Health* 2020(1), 8516105. 10.1155/2020/8516105 32565841 PMC7291314

[CIT0047] Sunder, J., 2016, ‘Effect of Morinda citrifolia in growth, production and immunomodulatory properties in livestock and poultry: A review Dietary supplementation of mineras for increasing the production of Livestock View project AICRP FMD View project’, *Journal of Experimental Biology and Agricultural Sciences* 4(3S), 249–265. 10.18006/2016.4(3S).249.265

[CIT0048] Taebipour, M.J., Dadras, H., Nazifi, S., Afsar, M. & Ansari-Lari, M., 2017, ‘Evaluation of blood monocyte and lymphocyte population in broiler chicken after vaccination and experimental challenge with Newcastle disease virus’, *Veterinary Immunology and Immunopathology* 190, 31–38. 10.1016/j.vetimm.2017.07.002 28778320

[CIT0049] The Uganda National Council for Science and Technology. (2020). *Uganda: UNCST issues guidelines for research during COVID-19 pandemic*, ClinRegs, viewed 10 August 2021, from https://clinregs.niaid.nih.gov/updates/full/43-uganda%3A-uncst-issues-guidelines-for-research-during-covid-19-pandemic.

[CIT0050] Viegi, L. & Vangelisti, R., 2011, ‘Toxic plants used in ethnoveterinary medicine in Italy’, *Natural Product Communications* 6(7), 999–1000. 10.1177/1934578x1100600719 21834243

[CIT0051] Wismer, T., 2015, ‘Advancements in diagnosis and management of toxicologic problems. Preventing unnecessary death by poisoning’, *Current Therapy in Avian Medicine and Surgery* 2016, 589–600. 10.1016/B978-1-4557-4671-2.00027-6

[CIT0052] Yuca, H., 2022, ‘Capsicum annuum L.’, in F.T. Gürağaç Dereli, M. Ilhan & T. Belwal (eds.), *Novel drug targets with traditional herbal medicines: Scientific and clinical evidence*, pp. 95–108, Springer International Publishing, Cham.

[CIT0053] Zirintunda, G., 2025, *Histological Sections of organs (heart, kidney and liver) of chicken treated with selected anthelmintic plant extracts, piperazine citrate, levamisole hydrochloride and PBS*, Figshare, London.

[CIT0054] Zirintunda, G., Kateregga, J., Nalule, S., Biryomumaisho, S., Omujal, F., Okwee-Acai, J. et al., 2025, ‘Extracts of *Carica papaya* L. and *Capsicum annuum* L. showed comparable efficacy to piperazine citrate and levamisole hydrochloride in treatment of poultry helminths’, *Beni-Suef University Journal of Basic and Applied Sciences* 14(1), 1–15. 10.1186/s43088-025-00607-z

